# Enhanced Thermal Shock Resistance of Porous Ca_2_Mg_2_Al_28_O_46_ Ceramic Filter via Nano-Sized ZrO_2_ Toughening

**DOI:** 10.3390/ma19050890

**Published:** 2026-02-27

**Authors:** Jianjun Shi, Hui Xu, Peixiong Zhang, Jingjing Liu, Enhui Wang, Bo Ren, Xinmei Hou

**Affiliations:** 1 Institute for Carbon Neutrality, University of Science and Technology Beijing, Beijing 100083, China; 2 Technical Support Center for Prevention and Control of Disastrous Accidents in Metal Smelting, University of Science and Technology Beijing, Beijing 100083, China; 3 School of Materials Science and Engineering, University of Science and Technology Beijing, Beijing 100083, China

**Keywords:** porous C_2_M_2_A_14_ ceramics, ZrO_2_ sol, thermal shock resistance, compressive strength, filtration efficiency

## Abstract

Porous Ca_2_Mg_2_Al_28_O_46_ (C_2_M_2_A_14_) ceramics are highly competitive candidates in the field of critical metal filtration due to their attractive non-metallic-inclusions removal capacity. However, the low mechanical strength and inadequate thermal shock resistance (TSR) of these materials restrict their further application. In this work, ZrO_2_-toughened C_2_M_2_A_14_-based porous ceramics are fabricated by using the polymer sponge replica method. Nano-sized ZrO_2_ particles derived from nano-ZrO_2_ sol are beneficial to enhance the mechanical properties and TSR of porous ceramics. The optimized porous C_2_M_2_A_14_ ceramics exhibit robust compressive strength (2.15 MPa), good residual strength ratio (66.4%) and excellent filtration efficiency in the reduction in total oxygen content (68.4%) by adding 3 wt% ZrO_2_ sol. These excellent comprehensive properties show that as-prepared porous C_2_M_2_A_14_ ceramics are promising candidate materials for application in the field of critical metal filtration.

## 1. Introduction

The rapid development of high-quality steel products requires efficient and multifunctional filtration materials that possess excellent filtration capacity, thermal stability, and mechanical robustness [1,2,3,4,5]. Various three-dimensional (3D) reticulated ceramics (e.g., SiC, Al_2_O_3_ and CaO) have been widely applied to remove the non-metallic inclusions (mainly Al_2_O_3_ inclusions) owing to their intricate pore network and high refractoriness [6,7,8,9,10,11,12]. However, the inadequate filtration capacity, poor thermal shock resistance (TSR) and being prone to hydration limit their broad applications. Recently, Ca_2_Mg_2_Al_28_O_46_ (C_2_M_2_A_14_)-based ceramics have shown greater potential for filtering Al_2_O_3_ inclusions because they contain non-free calcium oxide (CaO) that would react with Al_2_O_3_ inclusions [13,14,15,16]. Nevertheless, the relatively high thermal expansion coefficient (TEC, 8.92 × 10^−6^/°C) and low thermal conductivity (*κ*, 23.25 W·m^−1^·K^−1^, 25 °C) of C_2_M_2_A_14_ lead to the easy accumulation of thermal stress inside the material, deteriorating its thermal shock resistance [14]. Therefore, the promotion of the TSR of porous C_2_M_2_A_14_ ceramics is an urgent task.

Recently, the introduction of the second phase in porous ceramics has been demonstrated to be a versatile approach for enhancing their TSR [9,17,18]. The major reinforced phases for porous ceramics are mullite, magnesium aluminate spinel (MgAl_2_O_4_) [17,18,19] and silicon carbide (SiC) [20,21,22] and zirconia (ZrO_2_) [23,24,25]. Among various second phases, zirconia (ZrO_2_) has attracted widespread attention due to its enhanced fracture toughness through stress-induced tetragonal-monoclinic transformation, low thermal-expansion coefficient, and excellent high-temperature stability [26,27]. For instance, Chen et al. [28] prepared porous Al_2_O_3_-ZrO_2_-mullite composites with enhanced TSR through the transformation toughening of ZrO_2_ originating from the decomposition of ZrSiO_4_. Mao et al. [23] optimized the TSR of porous Si_3_N_4_-based ceramics by adding ZrO_2_ powder as the reinforcing phase. However, the direct introduction of these coarse and large-sized ZrO_2_ particles would inevitably lead to insufficient phase transformation of porous ceramic, limiting their further application. It has been demonstrated that incorporating nano-sized ZrO_2_ particles can substantially enhance the phase transformation degree [29]. However, the introduction of these nano-sized particles would inevitably lead to the agglomeration in the highly viscous ceramic slurry [30]. These present major challenges that must be tackled before extensive application.

Herein, we demonstrate a robust method for fabricating highly porous C_2_M_2_A_14_ ceramics with improved TSR by using ZrO_2_ sol as the reinforced composition. The homogeneously distributed nano-sized ZrO_2_ particles originating from ZrO_2_ sol effectively promote densification and phase transformation, thereby enhancing mechanical properties and TSR of porous ceramics. The as-prepared C_2_M_2_A_14_ ceramics demonstrate the integrated properties of relatively high porosity (81.12%), robust cold-compressive strength (2.15 MPa), good residual-strength ratio (66.4%) and excellent filtration efficiency (68.4%). These results demonstrate that the proposed C_2_M_2_A_14_ reticulated ceramics show great potential for application in molten steel purification.

## 2. Experimental

### 2.1. Materials and Fabrication Process of Porous C_2_M_2_A_14_ Ceramics

**Figure 1:**
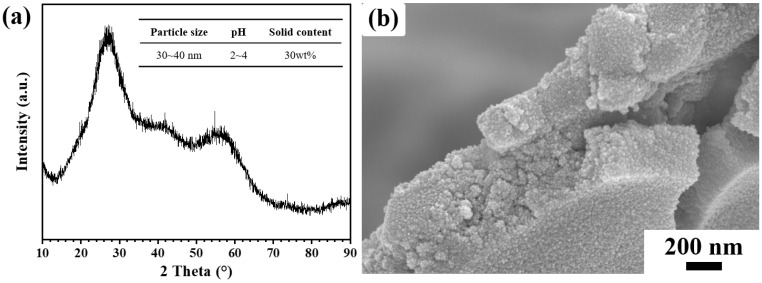
XRD patterns (**a**) and SEM images (**b**) of the ZrO_2_ sol.

The C_2_M_2_A_14_ powder (*d*_50_ = 20 μm) was synthesized by the solid phase reaction method. The synthetic procedure of C_2_M_2_A_14_ is as follows: The preparation process of C_2_M_2_A_14_ powders was as follows: Firstly, the mixture powders containing Al_2_O_3_, MgO and CaO according to the stoichiometric ratio were homogenized for 2 h at a rotating speed of 300 r/min using ethanol as the grinding medium. Subsequently, the mixture slurry was dried in an oven at 110 °C, ground and uniaxially pressed under 150 MPa to obtain green bodies with dimensions of 10 cm × 10 cm × 2 cm. These green bodies were calcined in a muffle furnace (KSL-1800X, Zhengzhou Kejing Electric Furnace Co., Ltd., Zhengzhou, China) at 1700 °C for 6 h before being dried at 110 °C for 24 h. Lastly, the calcined bulks were crushed and sieved to obtain C_2_M_2_A_14_ powder. The ZrO_2_ sol was purchased from Dezhou Jinghuo Technology Glass Co., Ltd., Dezhou, China, while polyurethane foam (PU, 15 PPI) was supplied by Wuxi Chenguang Refractory Materials Co., Ltd., Wuxi, China. Figure 1 shows the XRD patterns and SEM images of ZrO_2_ sol. It can be seen that the ZrO_2_ sol is amorphous, with its microstructure consisting of agglomerated spherical particles 20~30 nm in size. The solid content of the ZrO_2_ sol is 30 wt% and the pH value is 2~4. The carboxymethyl cellulose (CMC), ammonium lignosulfonate (AL) and polycarboxylate (WSM-M) were obtained from Shanghai Aladdin Biochemical Technology Co., Ltd., Shanghai, China, which were used as the thickener, binder and dispersant, respectively. The CMC, AL and WSM-M are introduced as external admixtures and are not incorporated into the matrix. They are collectively referred to as additives and are added to the ceramic slurry after mixing. In order to enhance the surface roughness of the PU foam and improve the adhesion of the ceramic slurry, the PU foam was pretreated by immersion in a 5 wt% NaOH solution at 25 °C for 24 h, based on relevant literature [31,32]. It was then washed with high-purity deionized water and dried.

**Figure 2:**
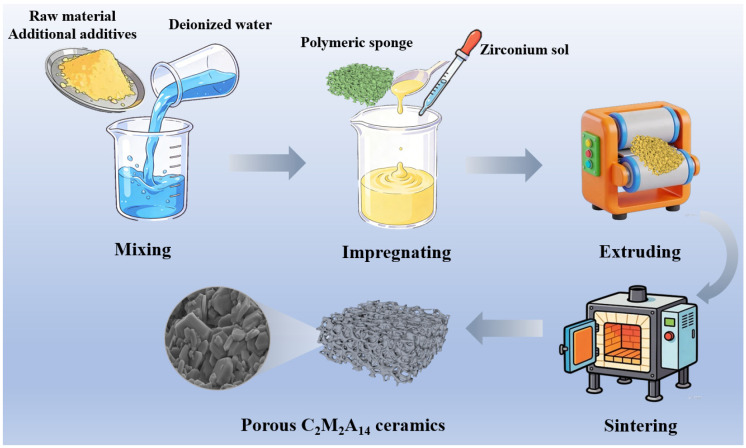
Schematic of the preparation process of porous C_2_M_2_A_14_ ceramics.

Figure 2 schematically depicts the fabrication procedure of the porous C_2_M_2_A_14_ ceramics. Firstly, the C_2_M_2_A_14_ ceramic powders, CMC, AL and WSM-M powders were mixed with deionized water and underwent high-speed mechanical stirring for 5 min to obtain the homogeneous ceramic slurry. The ZrO_2_ sol was added to the slurry, followed by an additional 3 min of high-speed stirring to ensure its uniform dispersion. Unless mentioned, the stirring speed used in this experiment is 2000 r/min. Subsequently, the pretreated PU foam (45 mm × 45 mm × 20 mm) was then immersed in the prepared ceramic slurry and pressed with tweezers to ensure complete immersion. Excess slurry was removed using a squeeze-roller device (SR-112, Hongjuyuan Jewelry Equipment Co., Ltd., Jinan, China). This immersion–squeezing cycle was repeated three times to obtain the porous C_2_M_2_A_14_ ceramics green body. The green body was first dried naturally for 24 h, then dried at 110 °C for another 6 h. Finally, the dried green body was calcined at 1600 °C for 2 h. According to the addition amount of ZrO_2_ sol (0, 1, 2, 3 and 4 wt%), the corresponding specimens were labeled as ZS0, ZS1, ZS2, ZS3 and ZS4 (Table 1), respectively.

### 2.2. Immersion Test

**Figure 3:**
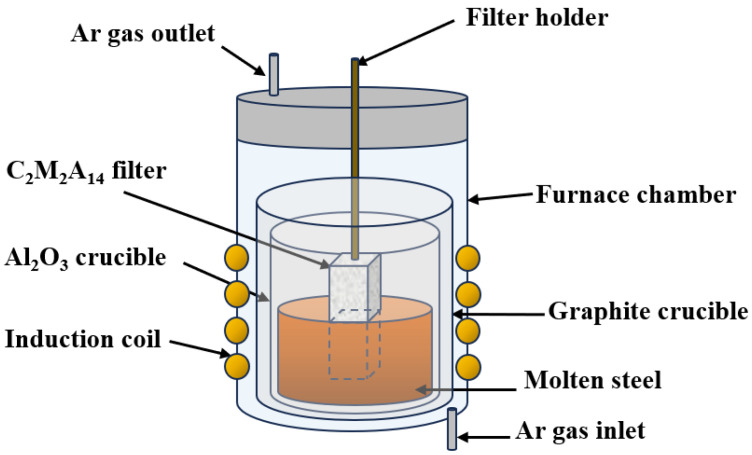
Schematic of the experimental setup for simulating molten steel immersion.

To evaluate the purification effect of porous C_2_M_2_A_14_ ceramics on molten steel, aluminum-killed steel was selected as the target melt, whose chemical composition is given in Table 2. The specific immersion test was carried out as illustrated in Figure 3. Firstly, a 200 g steel block was placed in ZrO_2_ crucible, and the porous ceramic was suspended above a block, then both were positioned together inside a graphite crucible. The assembly was placed in a vacuum-induction furnace (VIM-2, Nanjing Boyuntong Instrument Technology Co., Ltd, Nanjing, Jiangsu, China) and heated to 1600 °C at a rate of 15 °C/min under an argon atmosphere (The purity and flow rate of argon are 99.99% and 300 mL/min). Subsequently, the porous ceramic was immersed in the melt and held for 20 min when the steel was completely molten. Finally, the porous ceramic was removed, and the molten steel was cooled in a furnace. For each immersion test, three parallel specimens were tested, and the average value was taken.

### 2.3. Characterization

The rheological property of the C_2_M_2_A_14_ ceramic slurry at room temperature (25 °C) was measured using a stress-controlled rheometer (Haake Mars40, Karlsruhe, Germany) under continuous shear mode. The phase composition of the specimens was characterized by an X-ray diffractometer (XRD, D8 DISCOVER A25, Bruker AXS GmbH, Karlsruhe, Germany) with the Cu-Kα radiation (test 2θ angle range: 10~80°, scanning speed: 5°/min). Scanning electron microscopy (SEM, NanoSEM 450, Thermo Fisher Scientific, Hillsboro, OR, USA) equipped with an energy dispersive spectrometer (EDS, AMETEK EDAX, Mahwah, NJ, USA) was used to characterize the morphology and microstructure of the specimens. The bulk density and apparent porosity were determined via the Archimedes principle. The cold compressive strength (CCS) of the specimens was measured by the universal testing machine (ETM, MTS E45.105, Eden Prairie, MN, USA) according to the GB/T 1964-2023. Each performance was tested three times for the average value. The TSR of specimens was evaluated based on GB/T 16536-1996 via a water-cooling method [30,33]. The residual strength ratio (CCS after thermal shock testing/CCS before thermal shock testing) of the porous C_2_M_2_A_14_ ceramics was calculated to evaluate its TSR. The thermal-shock stability of the test specimens was averaged using three parallel specimens. The contents of [Al] and total oxygen (T.O.) in the steel were analyzed using an inductively coupled plasma spectrometer (ICP, Agilent 7800, Santa Clara, CA, USA) and oxygen-nitrogen analyzer equipment (HORIBA EMGA-830, Kyoto, Japan). Thermodynamic calculations were performed according to reference [34], and the mass fraction of ZrO_2_ was fixed at 1.25 wt%, while the mass fractions of CaO, Al_2_O_3_ and MgO were each varied from 0 to 1. The calculations were conducted at 1600 °C under a total pressure of 1 atm. The inclusion quantity statistics in steel were examined with a fully automatic inclusion analyzer (ARL iSpark 8860, Thermo Fisher Equipment Co., Ltd., Waltham, MA, USA). The removal efficiency of inclusions was evaluated based on Equation (1) by comparing the T.O. before and after immersion of molten steel.

(1) $$F=\frac{A-B}{A}\times 100\%$$

where *A* and *B* represent the contents of T.O. in steel before and after immersion using the porous C_2_M_2_A_14_ ceramics, respectively, and *F* is the removal efficiency of inclusions in steel.

## 3. Results and Discussion

### 3.1. Physical Properties of Porous C_2_M_2_A_14_ Ceramics

**Figure 4:**
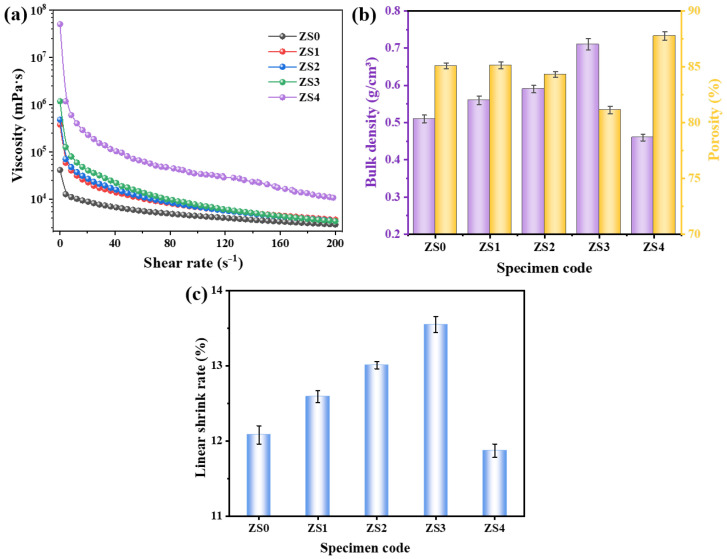
(**a**) Rheological properties, (**b**) bulk density and porosity and (**c**) linear shrinkage rate of as-prepared porous C_2_M_2_A_14_ ceramics containing various ZrO_2_ sol.

The rheological properties of the ceramic slurry critically influence the microstructure and performance of the final ceramic product. Figure 4a illustrates the rheological behavior of C_2_M_2_A_14_ ceramics slurries containing different additions of ZrO_2_ sol. From Figure 4a, the viscosity of all ceramic slurries decreases with increasing shear rate, exhibiting significant shear-thinning behavior. This characteristic facilitates rapid adhesion of the ceramic slurry to the PU foam scaffold surface during immersion. Furthermore, the viscosity of the ceramic slurry increases with the increase in ZrO_2_ sol addition, which is due to the enhanced interaction between the ZrO_2_ sol network that increases the resistance to flow within the slurry. For all specimens, the slurry with 3 wt% ZrO_2_ sol addition demonstrates the most favorable rheological properties. Figure 4b,c show the bulk density, porosity and linear shrinkage ratio of as-prepared porous C_2_M_2_A_14_ ceramics. Apparently, as the content of ZrO_2_ sol increases from 0 to 3 wt%, the bulk density rises from 0.51 to 0.71 g/cm^3^, while the porosity decreases from 85.06 to 81.12% (Figure 4b). This phenomenon is mainly attributed to the fact that nano-sized ZrO_2_ particles have an extremely high specific surface area and chemical activity, promoting the sintering of ceramic particles. However, as the ZrO_2_ sol further increases to 4 wt%, the bulk density decreases to 0.46 g/cm^3^, which is due to the poor rheological properties and relatively high-volume expansion from the phase transformation of ZrO_2_. The linear shrinkage rate shown in Figure 4c increases first and then decreases and the specimen ZS3 possesses the highest linear shrinkage of 13.55%.

### 3.2. Phase Composition and Microstructure

**Figure 5:**
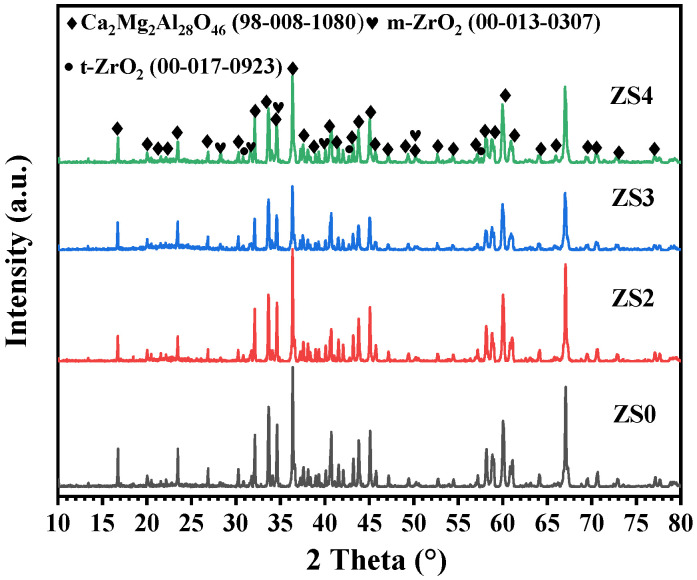
The XRD patterns of as-prepared porous C_2_M_2_A_14_ ceramics containing various ZrO_2_ sol additions.

Figure 5 shows the phase composition of as-prepared porous C_2_M_2_A_14_ ceramics with different ZrO_2_ sol additions. For specimen ZS0, the C_2_M_2_A_14_ is identified as the main phase. In contrast, for the porous C_2_M_2_A_14_ ceramics containing various ZrO_2_ sol additions (ZS2~ZS4), the C_2_M_2_A_14_, m-ZrO_2_ and a small amount of t-ZrO_2_ phase phases are detected, indicating ZrO_2_ sol has been successfully introduced into the porous C_2_M_2_A_14_ ceramics. With the increase in ZrO_2_ sol content, the intensity of m-ZrO_2_ diffraction peak does not change significantly, which may be due to the relatively low addition of ZrO_2_ sol.

**Figure 6:**
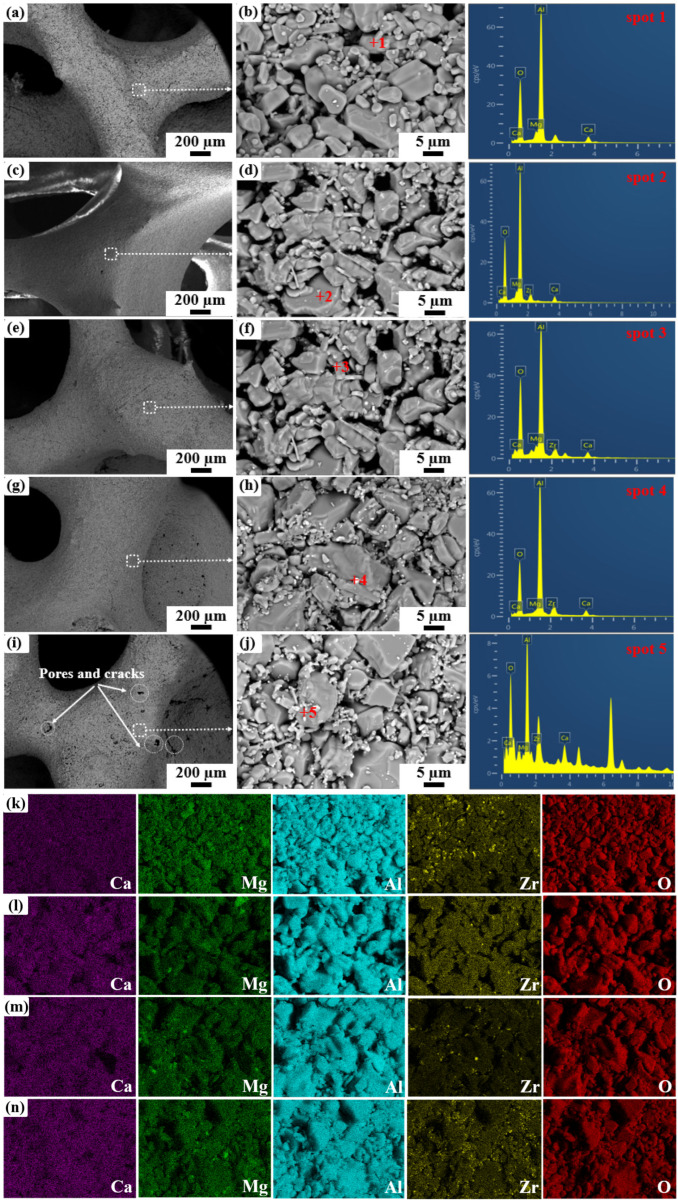
BSE images and EDS analysis of as-prepared porous C_2_M_2_A_14_ ceramics containing various ZrO_2_ sol after calcination at 1600 °C. (**a**,**b**): 0 wt%, (**c**,**d**): 1 wt%, (**e**,**f**): 2 wt%, (**g**,**h**): 3 wt%, (**i**,**j**): 4 wt%, (**k**–**n**): EDS mapping of porous C_2_M_2_A_14_ ceramics containing1, 2, 3 and 4 wt% ZrO_2_ sol. (The red number of +1~+5 is the position of EDS spot).

Figure 6 presents the microstructure of as-prepared porous C_2_M_2_A_14_ ceramics. For the specimen ZS0 (Figure 6a,b), the ceramic strut surface is composed of hexagonal plate-like grains and irregular particles packed together, with discernible interparticle pores. EDS result (spot 1) verified that these plate grains are mainly C_2_M_2_A_14_. With the addition of ZrO_2_ sol, as shown in Figure 6c–j, the strut becomes dense, which is attributed to the relatively high chemical activity of the nano-sized ZrO_2_ particles, promoting the sintering of the strut. The EDS analysis results from spot 2~5 indicate the presence of Zr in addition to Ca, Mg, Al and O, verifying that ZrO_2_ sol is successfully incorporated into the C_2_M_2_A_14_. It can be seen that the bright white nano-sized ZrO_2_ particles are uniformly dispersed on the plate-like C_2_M_2_A_14_ grains. Meanwhile, the number of bright white ZrO_2_ particles increases with increasing ZrO_2_ sol addition. The elemental mapping of specimen ZS1~ZS4 displayed in Figure 6k–n indicates that the elements Ca, Mg, Al and Zr are uniformly distributed in the scanned area, confirming the uniform dispersion of nano-sized ZrO_2_ particles. However, when the ZrO_2_ sol further increases to 4 wt%, some pores and cracks appeared on the strut surface. This may be due to excessive volume expansion from the phase transformation, leading to more formation of pores and cracks.

### 3.3. Thermal Shock Resistance

**Figure 7:**
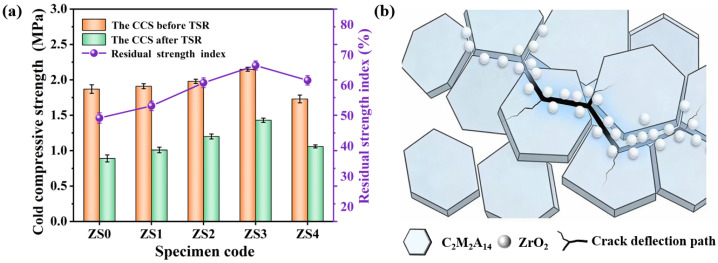
(**a**) The CCS before and after thermal shocks and the residual strength ratio of porous C_2_M_2_A_14_ ceramics, (**b**) Schematic diagram of the mechanism of thermal shock stability improvement.

The impact of ZrO_2_ sol addition on the CCS and TSR of porous C_2_M_2_A_14_ ceramics is presented in Figure 7. From Figure 7a, the CCS firstly increased and then decreased with the increasing of ZrO_2_ sol addition, which is consistent with the bulk density and porosity of specimens. The residual strength ratio (CCS after thermal shocks/CCS before thermal shocks) of porous C_2_M_2_A_14_ ceramics first increases and then decreases when the ZrO_2_ sol “µm” addition increases from 0 wt% to 4 wt%, and specimen ZS3 possesses the highest residual-strength ratio of 66.4%. Figure 7b illustrates the TSR mechanism of as-prepared porous C_2_M_2_A_14_ ceramics. Firstly, the hexagonal plate-like C_2_M_2_A_14_ grains can facilitate crack deflection and dissipate crack energy during thermal shock. Furthermore, the phase transformation of uniformly distributed nano-sized ZrO_2_ generates a few microcracks, which contribute to dispersing the propagation energy of the main crack. Meanwhile, owing to its high specific surface area, ZrO_2_ sol promotes the sintering of the strut, improving the ability to resist thermal shock. These combined effects would lead to the excellent TSR of as-prepared porous C_2_M_2_A_14_ ceramics. However, when the ZrO_2_ sol addition reaches 4 wt%, the excessive volume expansion during the phase transformation of ZrO_2_ leads to relatively more cracks, which inevitably deteriorate its TSR.

Table 3 compares the CCS and TSR of specimen ZS3 with other porous ceramics reported in the literature [11,30,35,36,37,38]. It can be seen that when the porosity is comparable, specimen ZS3 exhibits a high compressive strength of 2.15 MPa and relatively high residual strength retention of 66.4%. This is attributed to the sintering promotion effect of ZrO_2_ sol, the cross-stacked hexagonal platelet structure characteristics and the phase-transformation toughening effect of nano-sized ZrO_2_ particles. Unlike irregular ceramic particles such as Al_2_O_3_, MgO, ZrO_2_, SiC and MgAl_2_O_4_, the interlock hexagonal plate-like skeletal structure facilitates crack deflection and dissipates crack energy. Furthermore, the microcracks generated through the phase transformation between m-ZrO_2_ and t-ZrO_2_ are also beneficial for absorbing stress and dissipating crack energy [39,40,41,42].

### 3.4. Filter Performance

**Figure 8:**
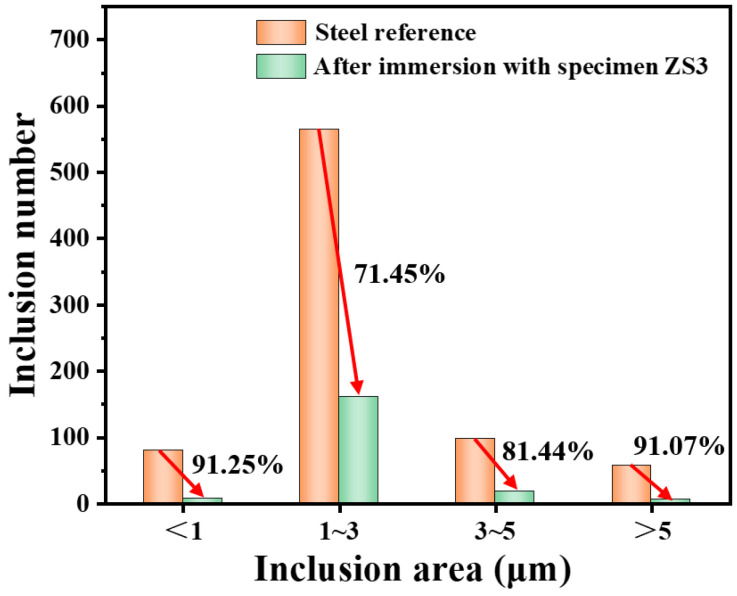
Inclusions in steel before and after immersion with specimen ZS3. (The red arrow indicates descending direction).

Considering both mechanical strength and TSR, specimen ZS3 is selected to evaluate its filter performance. Figure 8 displays the size and quantity of inclusions per 20 mm^2^ scanned area in the steel before and after immersion tests using the specimen ZS3. It can be seen that the number of inclusions smaller than 1 μm, 1~3 μm, 3~5 μm and those larger than 5 μm in the impregnated steel is significantly reduced compared with those in unimpregnated steel. The removal efficiencies for those inclusions are calculated to be 91.25%, 71.45%, 81.44% and 91.07%, respectively, indicating an effective removal capability for various sizes of inclusions. Meanwhile, the content of Al and total oxygen also decreases markedly from 0.251 and 0.005 to 0.101 and 0.00158 after immersion with specimen ZS3 (Table 4), which proves that Al_2_O_3_ inclusions are effectively removed.

**Figure 9:**
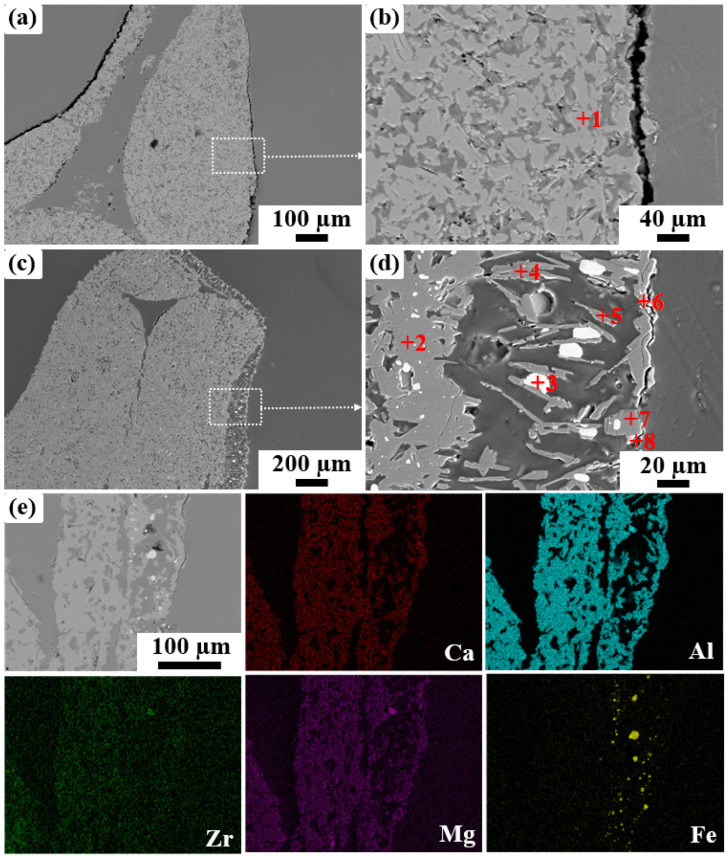
BSE images and mapping analysis of the specimen ZS3 after immersion. (**a**,**b**) the specimen ZS3 before immersion, (**c**,**d**) the specimen ZS3 after immersion, (**e**) the mapping of specimen ZS3 after immersion. (The red number of +1~+8 is the position of EDS spot).

**Figure 10:**
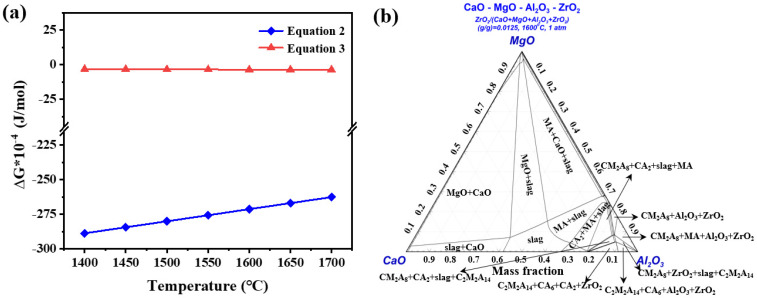
(**a**) Gibbs free energy of Equations (2) and (3) and (**b**) liquid phase region diagram for the reaction between C_2_M_2_A_14_ and molten steel at 1600 °C.

In order to clarify removal mechanism of the specimen ZS3, the cross-sectional microstructure of ZS3 before and after immersion is analyzed and the results are shown in Figure 9 and Table 5. Before immersion, the ceramic strut of specimen ZS3 consists of interlocked plate-like grains (Figure 9a,b), which are composed mainly of Ca, Mg, Al and O, with a small amount of Zr, confirming that they are ZrO_2_-toughened C_2_M_2_A_14_ (spot 1~2). After immersion, a reaction layer with an approximate thickness of 100 μm is formed (Figure 9c,d). For the reaction layer, the white particles primarily contain Fe (spot 3) and the elongated plate-like regions mainly contain Al, Ca and O, which correspond to the residual condensed Fe and mixtures of CaAl_12_O_19_ and CaAl_4_O_7_ (spot 4~5) inclusions. The irregular particles near the steel side consist mainly of Ca, Al, Mg and O, and are inferred to be mixtures of MgAl_2_O_4_, Al_2_O_3_, CaAl_4_O_7_ and CaAl_2_O_4_ (spot 6~8). This is consistent with the elemental mapping distribution in Figure 9e. Based on the above results, we infer that the removal mechanism of specimen ZS3 can be divided into two aspects: physical interception and chemical adsorption. For physical interception, the rough surface of the ceramic strut, created by the stacking of flake-like structure C_2_M_2_A_14_, increases the contact area with molten steel, thereby enhancing the inclusions interception probability. For chemical-reaction adsorption, under experimental conditions, C_2_M_2_A_14_ (Ca_2_Mg_2_Al_28_O_46_) would firstly react with [Mg], [Ca] and [O] in molten steel to form CaAl_4_O_7_, CaAl_2_O_4_ and MgAl_2_O_4_ (Equation (2)), and these three phases further form a liquid phase under smelting conditions, adsorbing the Al_2_O_3_ inclusions in molten steel. On the other hand, these Al_2_O_3_ inclusions can further react with CaAl_4_O_7_ and CaAl_4_O_7_ to form the CaAl_12_O_19_ phase (Equation (3)), resulting in the presence of CaAl_12_O_19_ at the interface between the C_2_M_2_A_14_ filter and molten steel, which further purifies the molten steel. Figure 10a shows the Gibbs free energy (ΔG) change in Equations (2) and (3) under thermodynamic conditions. The negative value of ΔG at 1600 °C indicates that these reactions are thermodynamically favorable, providing theoretical support for the proposed filtration mechanism. Figure 10b shows the ternary phase diagram of Al_2_O_3_ (ZrO_2_)-MgO-CaO phase diagram, which is employed to assess the phase transformation between C_2_M_2_A_14_ and molten steel at 1600 °C. The phase diagram result confirms the formation of new phases corresponding to Equations (2) and (3) in experimental conditions, along with the presence of a liquid phase in certain regions. These thermodynamic analyses are also consistent with the microstructural observations.

Ca_2_Mg_2_Al_28_O_46_ (s) + 4[Ca] + [Mg] + 5[O] = CaAl_2_O_4_ + 5CaAl_4_O_7_ + 3MgAl_2_O_4_ (2)

CaAl_2_O_4_ (s) + CaAl_4_O_7_ (s) + 9Al_2_O_3_ (s) = 2CaAl_12_O_19_ (s) (3)

## 4. Conclusions

This study systematically investigates the influence of ZrO_2_ sol on the rheological properties, physical properties and thermal-shock resistance as well as the inclusions-removal mechanism of porous C_2_M_2_A_14_ ceramics. The main conclusions can be drawn as follows:

(1) The incorporation of highly active ZrO_2_ sol promotes sintering, thereby enhancing compressive strength. Moreover, an appropriate amount of ZrO_2_ sol improves the TSR by generating microcracks via phase transformation. These cracks can facilitate crack deflection and crack dissipation, which enhances thermal shock stability.
(2) The optimized porous ZS3 ceramics exhibit a high compressive strength of 2.15 MPa and an excellent residual-strength ratio of 66.4%. Owing to the synergistic effect of physical interception and chemical reaction, the as-prepared porous C_2_M_2_A_14_-based ceramics achieve a high removal efficiency of 68.4% in total oxygen content. Given these superior properties, as-prepared porous C_2_M_2_A_14_ ceramic is a promising candidate for molten-metal filtration applications.

## Figures and Tables

**Table 1:** Experimental formulations of the porous C_2_M_2_A_14_ ceramics (wt%).

Specimen Code		ZS0	ZS1	ZS2	ZS3	ZS4
Raw materials	C_2_M_2_A_14_	72	72	72	72	72
	Deionized water	28	27	26	25	24
	ZrO_2_ sol	0	1.0	2.0	3.0	4.0
Additives	CMC (extra)	0.5	0.5	0.5	0.5	0.5
	AL (extra)	1.0	1.0	1.0	1.0	1.0
	WSM-M (extra)	0.3	0.3	0.3	0.3	0.3

**Table 2:** Chemical compositions of the aluminum-killed steel.

Elements	C	Si	Mn	S	Al	Ca	Mg	N	Fe
Contents/wt%	0.004	0.03	0.10~0.20	0.01	0.034	0.0017	0.0008	0.0021	Residual amount

**Table 3:** The performance comparison of the specimen ZS3 with other reported works.

Porous Ceramics	Preparation Method	Porosity (%)	CCS (MPa)	Thermal Shock Conditions	Residual Strength Ratio (%)	References
Porous MgAl_2_O_4_-MgO ceramics	Template replication method	78.25	0.85	1100 °C, Air cooling cycle 3 times	54.12	[35]
Porous Al_2_O_3_-ZrO_2_ ceramics	Template replication method	80.49	1.02	1100 °C, water cooling cycle 3 times	56.86	[36]
Porous corundum-spinel ceramics	Template replication method	/	0.53	1100 °C, Air cooling cycle 3 times	62.3	[30]
Porous Al_2_O_3_ ceramics	Template replication method	81	0.74	1100 °C, water cooling cycle 3 times	61	[37]
Porous SiC ceramics	Template replication method	87.5	0.38	1100 °C, water cooling cycle 3 times	44.75	[38]
Porous ZS3 ceramics	Template replication method	81.12	2.15	1100 °C, water cooling cycle 3 times	66.4	This work

**Table 4:** Chemical compositions of the steel specimens after immersion testing.

Steel Specimen	Al (wt%)	T.O. (wt%)
Steel reference	0.251	0.005
After immersion with specimen ZS3	0.101	0.00158

**Table 5:** The element content at points in Figure 9 (at%).

Point	Ca	Mg	Al	O	Zr	Fe	Possible Phase
1	3.05	2.97	40.52	52.88	0.58	/	Ca_2_Mg_2_Al_28_O_46_
2	3.09	2.98	39.88	53.53	0.41	0.11	Ca_2_Mg_2_Al_28_O_46_
3	0.07	/	0.09	1.81		98.03	Fe
4	4.38	0.14	48.15	47.03	0.08	0.22	CaAl_12_O_19_ and CaAl_4_O_7_
5	3.99	0.38	47.79	47.73	0.11	/	
6	0.19	11.58	42.39	45.40	0.13	0.31	Al_2_O_3_ and MgAl_2_O_4_
7	0.13	0.09	46.80	52.91	0.07	/	Al_2_O_3_
8	4.22	0.08	46.65	48.61	0.21	0.23	Al_2_O_3_, CaAl_4_O_7_ and CaAl_2_O_4_

## Data Availability

The original contributions presented in this study are included in the article. Further inquiries can be directed to the corresponding authors.

## Abbreviations

The abbreviations table.

Abbreviations	Technical Term
TEC	Thermal expansion coefficient
TSR	Thermal shock resistance
*κ*	Thermal conductivity
CMC	Carboxymethyl cellulose
AL	Ammonium lignosulfonate
WSM-M	Polycarboxylate
PU	Polyurethane foam
XRD	X-ray diffractometer
SEM	Scanning electron microscopy
EDS	Energy dispersive spectrometer
CCS	Cold compressive strength
T.O.	Total oxygen
ΔG	Gibbs free energy
CA_2_	CaAl_4_O_7_
CA	CaAl_2_O_4_
CA_6_	CaAl_12_O_19_
C_2_M_2_A_14_	Ca_2_Mg_2_Al_28_O_46_
CM_2_A_8_	CaMg_2_Al_16_O_27_
MA	MgAl_2_O_4_
